# Assessing the appropriateness of helicopter emergency medical services for non-traumatic emergencies in a medically underserved rural area, Japan

**DOI:** 10.1371/journal.pone.0353451

**Published:** 2026-07-09

**Authors:** Shohei Matsubara, Ken-ei Sada, Masataka Kudo, Sho Sasaki, Atsushi Morizane, Koshi Kajihara, Hidenao Okubo, Kosuke Hamagawa, Toshimi Mizobuchi, Toshikazu Yabe, Yuichi Saisaka, Kimiaki Tanaka, Narufumi Suganuma

**Affiliations:** 1 Department of Clinical Epidemiology, Kochi Medical School, Kochi University, Nankoku, Japan; 2 Department of Internal Medicine, Oida Hospital, Sukumo, Japan; 3 Department of Internal Medicine, Inan Hospital, Tosashimizu, Japan; 4 Department of General Internal Medicine, Iizuka Hospital, Fukuoka, Japan; 5 Department of General Medicine, Iwase Satellite for Teaching And Research (STAR), Fukushima Medical University, Fukushima, Japan; 6 Center for Innovative Research for Communities and Clinical Excellence (CiRC2LE), Fukushima Medical University, Fukushima, Japan; 7 Section of Clinical Epidemiology, Department of Community Medicine, Graduate School of Medicine, Kyoto University, Kyoto, Japan; 8 Department of Emergency Medicine, Kochi Health Sciences Center, Kochi, Japan; 9 Sukumo Fire Department, Hata Western Fire Department Union, Sukumo, Japan; 10 Department of Internal Medicine, Otsuki Hospital, Otsukicho, Japan; 11 Department of Internal Medicine, Shimanto City Hospital, Shimanto, Japan; 12 Department of Surgery, Inan Hospital, Tosashimizu, Japan; 13 Department of Cardiology, Kochi Prefectural Hata Kenmin Hospital, Sukumo, Japan; 14 Department of Surgery, Oida Hospital, Sukumo, Japan; 15 MEDi Center, Kochi University, Kochi, Japan; University of Alabama at Birmingham, UNITED STATES OF AMERICA

## Abstract

**Background:**

Helicopter emergency medical services (HEMS) are essential in trauma care, but their role in non-traumatic emergencies remains unclear, particularly in medically underserved rural areas where long total prehospital times and limited specialty care access complicate emergency response.

**Objectives:**

To evaluate the appropriateness of HEMS dispatches in non-trauma emergencies by assessing undertriage and overtriage based on clinical necessity and total prehospital time in rural western Kochi Prefecture, Japan.

**Methods:**

We conducted a retrospective cohort study of adult patients (≥18 years) requiring emergency transport for moderate to severe non-traumatic conditions during HEMS operational hours from 2017 to 2021. HEMS necessity was classified as “absolute” (treatment unavailable at local core hospitals) or “relative” (critical condition benefiting from shorter transport). Appropriateness was defined as concordance between actual transport modality and predefined necessity criteria, with undertriage and overtriage determined by comparing the actual primary HEMS dispatch requests against these criteria and regional total prehospital times.

**Results:**

Of the 2,312 eligible patients, 63 received primary HEMS dispatches and 2,249 ground EMS (GEMS). Among HEMS dispatches, 56 of 63 (88.9%) were overtriaged, while seven (11.1%) were appropriately triaged. Among GEMS transports, 248 of 2,249 (11.0%) retrospectively met HEMS criteria, yielding an 11.0% undertriage rate. Overtriaged cases included patients lacking relative necessity criteria or total prehospital time advantage.

**Conclusions:**

In medically underserved rural regions, clinical severity alone may be insufficient for determining HEMS necessity in non-traumatic emergencies. Incorporating total prehospital time advantage and regional infrastructure consideration may refine dispatch decision-making and improve HEMS utilization appropriateness.

## Introduction

Helicopter emergency medical services (HEMS) are costly, specialized resources intended for patients with severe trauma or time-critical emergencies [1]. To ensure efficient use, appropriate dispatch decisions and accurate triage are essential. According to a 2021 joint position statement, HEMS should be activated when [2] (1) local facilities cannot provide necessary care, (2) rapid transport is necessary for time-sensitive interventions, or (3) geographical or logistical barriers hinder timely access to care. These criteria aim to reduce mortality and morbidity from undertriage and avoid excessive costs from overtriage.

Although HEMS has been shown to improve outcomes in trauma cases [3–8], its benefits in non-traumatic emergencies remain unclear. In trauma, HEMS triage is largely guided by mechanism of injury and anatomical severity, and the benefit of rapid transport and on-scene intervention is well established. In contrast, non-traumatic emergencies often present with nonspecific symptoms, fewer immediately apparent indications for invasive prehospital interventions, and greater uncertainty in severity assessment at the time of dispatch. Prior studies have produced mixed results, with some reporting no survival benefit and others reporting delays in treatment for non-trauma cases transported by HEMS [9,10]. Dispatch guidelines for non-trauma vary widely, and the appropriateness of HEMS use in these cases is less well defined, particularly in rural settings where total prehospital time and access to definitive care vary substantially.

Triage accuracy is typically evaluated by the rates of overtriage and undertriage. In one trauma study, an undertriage rate, defined as cancellation of HEMS despite actual need, is 4.3% of cases [11]. Overtriage rates are higher, ranging from 17% to 54% [11–13], often based on cases where HEMS was dispatched but later canceled. However, these assessments are limited by a focus on dispatched patients only, and data on those never considered for HEMS remain scarce. For non-traumatic emergencies, only one study has reported an overtriage rate (13.1%) [1], and the undertriage rate remains largely unexplored.

To address these gaps, we conducted a retrospective analysis of non-traumatic emergencies in a rural area of Japan. Our objectives were to describe real-world HEMS dispatch patterns and to evaluate the rates and determinants of overtriage and undertriage of primary HEMS dispatch by linking prehospital dispatch data with final hospital dispatch diagnoses.

## Materials and methods

### Study design and setting

We conducted a retrospective cohort study in the southwestern coastal region of Kochi Prefecture, Japan, using data from January 1, 2017, to December 31, 2021. This study incorporated data from fire department ground emergency medical services (GEMS) dispatch records, HEMS dispatch records, and medical records from receiving hospitals. The data were first accessed for research purposes on February 1, 2023. Authorized personnel may have had access to identifiable information during data extraction and record linkage; however, all data were anonymized before analysis, and the authors did not have access to identifiable information during the analytical phase.

This study was conducted in the Hata region, which spans 1,238 square kilometers, includes approximately 80,000 residents, and is located approximately 100 km from Kochi City, the prefecture’s main urban center. This region is officially designated by the Japanese Ministry of Health, Labour and Welfare as a *medically underserved area (hekichi iryo chiiki)* due to its low physician-to-population ratio (1.5 physicians per 1,000 residents), sparse population densities (< 50 persons/km² in most municipalities), and a high aging rate (≥40% of residents aged ≥65 years).

In Japan, EMS access begins with a call to the local fire department, which dispatches an ambulance and, when appropriate, requests HEMS. In Kochi Prefecture, HEMS requests for non-traumatic emergencies are not strictly triggered by a keyword-based dispatch system; rather, paramedics determine whether to request HEMS based on information available at the initial call and their on-scene assessment. Accordingly, paramedics can place either a primary HEMS request at the time of the initial call or a secondary request after patient contact. Kochi Prefecture operates a single HEMS helicopter with one emergency physician and one nurse. The HEMS base is located in Kochi City, where four tertiary emergency facilities receive most HEMS cases. Ground ambulances are staffed by emergency medical technicians, including paramedics, and do not carry physicians. Physician-staffed ground vehicles are not routinely available in the study region. For this study, the Hata region was divided into eight predefined medical service areas corresponding to existing municipal boundaries. These areas are covered by one main local core hospital, four secondary hospitals, and seven fire departments. The local core hospital provides 24-hour emergency care and inpatient management for common non-traumatic emergencies, including stroke, acute coronary syndrome, heart failure, pneumonia, and sepsis. Acute thrombolytic therapy and emergency coronary catheterization can be provided at the local core hospital, although selected advanced cases require transfer to tertiary emergency medical centers in Kochi City. Advanced tertiary-level interventions, including surgical management of Stanford type A aortic dissection, emergency revascularization for acute myocardial infarction requiring advanced management of three-vessel disease, and catheter-based intervention for pulmonary embolism, are centralized in tertiary emergency medical centers in Kochi City.

### Participants

Eligible patients were those who required emergency transport by EMS during HEMS operating hours and met the following inclusion criteria: (1) aged 18 years or older, (2) transported to either a local core hospital or a tertiary emergency medical center in Kochi City, and (3) non-traumatic presenting moderate to severe emergencies, as determined by the attending physician at the time of emergency transport based on the overall clinical presentation, including vital signs, symptoms, and the need for urgent medical evaluation. For the purposes of this analysis, HEMS operational hours were defined as 8:30 AM to 6:30 PM, consistent with standard daytime operations. Although actual HEMS availability varies seasonally according to sunset time, case-level adjustment for daylight conditions was not performed. We excluded cases meeting any of the following: (1) transport cancellation, defined as cases in which the patient was ultimately not transported to a hospital by either HEMS or ground EMS, (2) cardiopulmonary arrest upon initial contact, (3) terminal illness and (4) originating from Areas G or H, where no HEMS dispatches occurred during the study period, making empirical estimation of total prehospital time impossible. Patient severity at the time of dispatch was assessed using fire department records.

### Variables

Key variables extracted from fire department and HEMS dispatch records included age, sex, location of emergency, chief complaint, type of HEMS dispatch (primary/secondary), and total prehospital time. Total prehospital time was defined as the interval from the initial emergency call to arrival at the receiving hospital. We used total prehospital time rather than the conventional transport interval from scene departure to hospital arrival because our aim was to evaluate the appropriateness of the initial dispatch decision, that is, whether HEMS should have been requested at the time of the emergency call. This definition reflects the overall time consequence of choosing HEMS or GEMS at the dispatch stage. After cross-matching these records with hospital data, we extracted additional variables: initial vital signs and final diagnoses. Final diagnoses were identified through medical record review and were subsequently classified into diagnostic categories aligned with the International Classification of Diseases, 11th edition (ICD-11).

### Primary outcome and appropriateness criteria

The primary outcome was the appropriateness of primary HEMS dispatch, assessed retrospectively and independently by two emergency physicians, with disagreements resolved by consensus. Both reviewers were board-certified specialists in emergency medicine and were independent of the HEMS dispatch process. In the absence of universally accepted criteria for defining the appropriateness of HEMS dispatch in non-traumatic emergencies, appropriateness in this study was operationally defined based on a combination of total prehospital time efficiency, clinical severity, and the anticipated need for advanced medical interventions. This definition was informed by previously published studies and existing dispatch frameworks for HEMS utilization [9–11], although we acknowledge that this approach relies partly on clinical judgment and may be subject to residual subjectivity.

We classified appropriateness using the following definitions:

- Absolute necessity: Conditions requiring advanced, time-critical interventions that are unavailable at local core hospitals, for which HEMS dispatch is considered necessary regardless of total prehospital time. Because the availability of definitive interventions differs by region, our classification of ‘absolute’ versus ‘relative’ necessity was designed as a context-specific operational definition for this medically underserved area. ‘Absolute necessity’ was restricted to conditions for which definitive treatment is typically unavailable at local core hospitals and requires transfer to tertiary centers. In this study, absolute necessity was predefined and limited to the following diagnoses: Stanford type A aortic dissection; acute myocardial infarction requiring emergent revascularization for three-vessel disease; and pulmonary embolism requiring catheter-based intervention.
- Relative necessity: Case in which HEMS provided a total prehospital time advantage and either:
- The condition benefits substantially from early treatment.
- The patient was considered clinically unstable upon arrival at the receiving hospital, including hemodynamic instability (systolic blood pressure < 90 mmHg or mean arterial pressure < 65 mmHg) or respiratory instability (oxygen saturation < 90%).

Relative necessity was evaluated for specific pre-specified diagnoses (full list in S1 Table) based on previous literature and expert consensus.

To evaluate relative necessity, we compared mean historical total prehospital times between HEMS and GEMS using trauma transport data from the same region. Mean values were calculated separately for each combination of departure area and receiving facility. For each patient, the comparison was based on the receiving hospital to which the patient was actually transported. HEMS was considered to provide a time advantage when the mean historical total prehospital time for HEMS was shorter than that for GEMS within the same departure-area and receiving-facility combination. We did not simulate patient-level GEMS-only or HEMS transport times. In actual HEMS operations, ground EMS may assess the patient, provide initial care, and prepare the patient for transport, or transport the patient to a rendezvous point before handover to HEMS. However, because our aim was to evaluate the system-level time consequence of using HEMS, total prehospital time was used as the primary time measure.

### Definition of overtriage and undertriage

Overtriage: Cases in which HEMS was requested (primary dispatch), but the patient did not meet criteria for absolute or relative necessity.

Undertriage: Cases in which GEMS-only transport was used, but the patient *did* meet HEMS criteria (absolute or relative)

Importantly, we defined these metrics using final diagnoses and retrospective physician assessment, acknowledging that prehospital impressions may differ.

### Statistical methods

Descriptive statistics were used to summarize patient characteristics and diagnoses. Continuous variables are presented as mean and standard deviation (SD), and categorical variables as counts and percentages.

Diagnoses with ≥100 cases were reported individually; others were aggregated into broader ICD-11 diagnostic categories. Overtriage and undertriage rates were calculated using:

Overtriage rate = (Patients with primary HEMS dispatch who did *not* meet necessity criteria) / (Total primary HEMS dispatches)
Undertriage rate = (Patients who met necessity criteria but were transported by GEMS) / (Total GEMS-only cases)
All statistical analyses were performed using Stata software (version 17.0; StataCorp, College Station, TX, USA).

### Ethics statement

This study was approved by the Ethics Committee of Kochi University (Approval No. 2022−101) and conducted in accordance with the Declaration of Helsinki and the Ethical Guidelines for Medical and Health Research Involving Human Subjects in Japan. All data were anonymized prior to analysis. Due to the retrospective nature of the study, the requirement for written informed consent was waived using an opt-out approach.

## Results

### Participants

**Fig 1:**
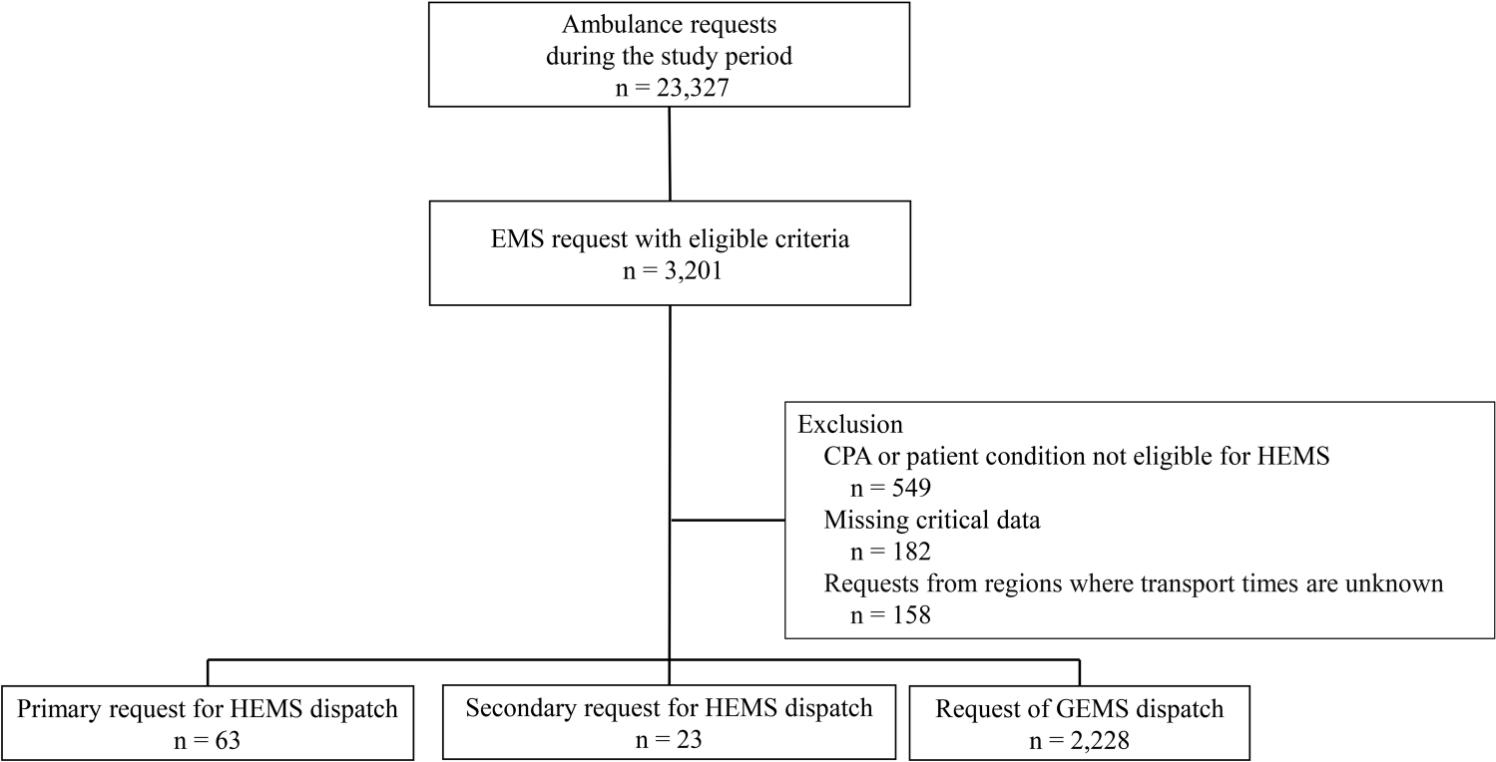
Flowchart showing all missions and the inclusion of patients. CPA, cardiopulmonary arrest; EMS, emergency medical service; GEMS, ground emergency medical service; HEMS, helicopter emergency medical service.

Of the 23,327 ambulance requests made during the study period, 3,201 met the inclusion criteria. After excluding 889 patients based on the exclusion criteria, 2,312 patients were enrolled in this study (Fig 1). Among them, 63 underwent primary HEMS dispatch, 23 secondary HEMS, and 2,228 GEMS.

The mean age was 77.4 years (SD, 13.2), and 1,028 of 2,312 patients (44.5%) were females. Shock vital signs were observed in 133 of 2,093 patients (6.4%), and hypoxemia in 66 of 1,995 patients (3.3%) (Table 1). The number of ambulance requests by transport method in each area is listed in S2 Table.

**Table 1:** Patient characteristics.

	Total (n = 2312)	Primary request for HEMS dispatch (n = 63)	Secondary request for HEMS dispatch (n = 21)	Request for GEMS dispatch (n = 2228)
Age, mean (SD), years	77.4 (13.2)	74.5 (14.9)	71.7 (16.6)	77.6 (13.1)
Female, n (%)	1028/2312 (44.5)	22/63 (34.9)	8/21 (38.1)	998/2228 (44.8)
Low blood pressure, n (%)	133/2093 (6.4)	2/62 (3.2)	1/21 (4.7)	130/2010 (6.5)
Hypoxemia, n (%)	66/1995 (3.3)	1/59 (1.7)	0/18 (0)	65/1918 (3.4)
Severe disease, n (%)	1077/2312 (46.5)	41/63 (65.1)	16/21 (76.2)	1020/2228 (45.8)

Low blood pressure: systolic blood pressure <90 mmHg or mean arterial pressure <65 mmHg.

Hypoxemia: SpO2 < 90% at a hospital.

GEMS, ground emergency medical service; HEMS, helicopter emergency medical service.

SD, standard deviation.

### Final diagnoses

The most frequent diagnoses across all groups were: cerebral infarction (334 patients, 14.4%), bacterial pneumonia (250 patients, 10.8%), intracerebral hemorrhage (132 patients, 5.7%), acute pyelonephritis (126 patients, 5.5%), and heart failure (107 patients, 4.6%) (Table 2). In the primary HEMS dispatch group, top diagnosis were cerebral infarction (14 patients, 22.2%), myocardial infarction (seven patients, 11.1%), and intracerebral hemorrhage (five patients, 7.9%). In the secondary HEMS group, cerebral infarction (six patients, 28.6%) and epilepsy (five patients, 23.8%) were common. In the GEMS group, cerebral infarction (314 patients, 14.1%) and bacterial pneumonia (246 patients, 11.0%) predominated.

**Table 2:** Final diagnosis of enrolled patients.

Disease name		Number (%) of patients
Specific diseases	Cerebral infarction	334 (14.4)
	Bacterial pneumonia	250 (10.8)
	Intracerebral hemorrhage	132 (5.7)
	Acute pyelonephritis	126 (5.5)
	Heart failure	107 (4.6)
	Dehydration	100 (4.3)
Disease categories	Diseases of the digestive system	290 (12.5)
	Diseases of the circulatory system other than cerebral infarction, intracerebral hemorrhage, and heart failure	266 (11.5)
	Diseases of the nervous system	99 (4.3)
	Diseases of the respiratory system other than bacterial pneumonia	89 (3.8)
	Diseases of the ear or mastoid process	86 (3.7)
	Symptoms, signs or clinical findings, not elsewhere classified	83 (3.6)
	Endocrine, nutritional or metabolic diseases other than dehydration	70 (3.0)
	Certain infectious or parasitic diseases	60 (2.6)
	Injury, poisoning or certain other consequences of external causes	56 (2.4)
	Neoplasms	48 (2.0)
	Diseases of the musculoskeletal system or connective tissue	46 (2.0)
	Diseases of the skin	21 (0.9)
	Diseases of the genitourinary system other than acute pyelonephritis	21 (0.9)
	Mental, behavioral or neurodevelopmental disorders	13 (0.6)
	Disease of the blood or blood-forming organs	12 (0.5)
	Undiagnosed	3 (0.1)

### Necessity for HEMS dispatch

A total of 1,075 patients had final diagnoses that were included in the predefined list of conditions eligible for relative HEMS necessity. However, only 253 of these patients fulfilled the full criteria for relative necessity, which required both an eligible diagnosis and a shorter estimated total prehospital time via HEMS compared to GEMS from their respective locations.

Additionally, two patients met the criteria for absolute necessity, defined as Stanford type A aortic dissection requiring care at a tertiary center. Estimated total prehospital times (S3 Table) showed that only Area D had shorter average total prehospital time with HEMS than with GEMS.

### Appropriateness of HEMS dispatch

Among 63 primary HEMS requests, seven (11.3%) were deemed appropriate based on the necessity criteria.

Among the 2,249 GEMS transports, 248 patients (11.0%) were retrospectively determined to have met the criteria for HEMS dispatch, including two with absolute necessity and 246 with relative necessity. Notably, all relative-necessity cases originated from Area D, the only region in which HEMS was estimated to provide a total prehospital time advantage (Table 3). These patients were considered undertriaged, defined as cases in which HEMS was indicated but not utilized, resulting in an undertriage rate of 11.0%. Although the study period included the COVID-19 pandemic, none of the undertriaged cases involved COVID-19 or other febrile conditions subject to pandemic-related transport restrictions. Common conditions among undertriaged patients included: cerebral infarction (60 patients, 24.2%), heart failure (33 patients, 13.3%) and intracerebral hemorrhage (26 patients, 10.5%) (S4 Table). In contrast, 56 of 63 patients transported via primary HEMS were classified as overtriaged, yielding an overtriage rate of 88.9%. Among these, 19 patients (33.9%) had neither a condition eligible for relative necessity nor evidence of time savings with HEMS. Diagnoses in this subgroup included bacterial pneumonia, hypokalemia, chronic/acute subdural hematoma, viral infections, cancer, and metabolic/toxic causes. The remaining 37 patients had potentially eligible conditions but were judged to be overtriaged because the estimated total prehospital time was longer with HEMS than with GEMS. Their diagnoses were cerebral infarction (13 patients, 35.1%), myocardial infarction (six patients, 16.2%), intracerebral hemorrhage (five patients, 13.5%), and the other conditions (S5 Table). Overtriaged cases by region were: Area A (11 patients, 19.6%), B (17 patients, 30.4%), C (23 patients, 41.1%), D (one patient, 1.8%), E (three patients, 5.4%), F (one patient, 1.8%).

**Table 3:** Distribution of primary dispatch and necessity for HEMS.

		Primary request for HEMS dispatch	
		+	–
Necessity for HEMS	+	7	248
	–	56	2001

HEMS, helicopter emergency medical service.

## Discussion

This study evaluated the appropriateness of primary HEMS requests for non-traumatic emergencies in a medically underserved rural area. We found that undertriage occurred exclusively in one area (Area D) where HEMS provided a shorter total prehospital time than GEMS, while overtriage was common and frequently involved cases that neither met relative necessity criteria nor benefited from shorter HEMS transport.

Total prehospital time appears to be the most critical factor in HEMS decisions for non-trauma cases. The American College of Surgeons (ACS) recommends an undertriage threshold of ≤5% for trauma [14]. However, no such standard exists for non-trauma. Unlike trauma, where on-site interventions significantly affect outcomes, non-trauma cases rarely benefit from such care. Thus, reaching a facility capable of providing definitive treatment quickly is crucial. In this study, all undertriaged patients were in Area D, the only region where HEMS had a time advantage, suggesting that location-based time differences are essential for minimizing undertriage.

To improve the overtriage rate, it is essential to consider the request location. Currently, no established benchmark exists for overtriage in non-traumatic emergencies involving HEMS. The ACS guidelines recommend an overtriage rate of 25–35% for traumatic emergencies [14]. Previous studies reported HEMS dispatch cancellation rates–a form of overtriage ranging from 17% to 54% [11–13], which is much lower than the rate observed in our study. In non-traumatic emergencies, dispatch decisions often rely on subjective symptoms, such as severe pain (e.g., headache, chest pain, or abdominal pain), making triage heavily dependent on on-scene judgment. In fact, 34% of patients in the present study were in a condition that did not necessitate a HEMS request, highlighting the inherent difficulty of accurate severity assessment at the time of dispatch. Because dispatch-time information is often limited, we defined necessity based on hospital-arrival severity and final diagnoses. Although this approach involves hindsight bias, it allowed us to evaluate whether patients who ultimately proved to have severe conditions received a transport modality consistent with their clinical needs. Notably, among patients classified as overtriaged, a substantial proportion did not demonstrate a total prehospital time advantage with HEMS compared with GEMS. This finding suggests that clinical presentation alone may be insufficient to identify cases in which HEMS provides additional benefit in this specific regional context. Accordingly, in the setting where local core hospitals are capable of managing most non-traumatic conditions, incorporating estimates of total prehospital time may complement clinical assessment when refining HEMS dispatch decision-making.

Our study has several strengths. First, while most previous studies assessing HEMS triage evaluated only HEMS dispatch cases [1,12,15–17], thus making it impossible to assess the undertriage rate, our study integrated both GEMS and HEMS dispatch data. This design enabled simultaneous evaluation of both undertriage and overtriage in non-traumatic emergencies. Second, previous studies typically assessed the necessity of HEMS dispatch based on final diagnoses, clinical severity, and duration of hospitalization in traumatic emergencies [1,11,18,19]. In contrast, our study focused on non-traumatic emergencies and considered estimated total prehospital time advantage as an additional contextual factor alongside clinical severity, rather than as a sole determinant of HEMS necessity.　Importantly, among undertriaged cases in the present study, we did not identify conditions in which immediate airway management or medication administration would have been clearly time-critical, such as severe asthma or acute respiratory distress syndrome. Instead, undertriaged patients predominantly presented with cerebrovascular and cardiovascular conditions, for which definitive management depends largely on timely access to specialized in-hospital care. This observation provides insight into the types of non-traumatic emergencies in which HEMS may offer potential value in this specific rural setting.

Our study has some limitations. First, detailed physiological variables at the time of dispatch, particularly respiratory rate, were not consistently available, precluding calculation of standardized severity scores such as National Early Warning Score or quick Sequential Organ Failure Assessment [20,21]. Instead, clinical instability was assessed using available indicators of hemodynamic or respiratory instability; however, oxygen saturation does not fully capture abnormalities in ventilation or respiratory effort, which may have limited severity assessment. Second, total prehospital time was estimated using region-based averages from traumatic emergency data, and individual patient-level variability in transport conditions could not be fully accounted for. In actual HEMS missions, the extent of GEMS involvement may vary, including patient assessment, initial care, preparation for transport, and transport to a rendezvous point before handover to HEMS. Although total prehospital time reflects the system-level time from emergency call to hospital arrival, our area-level historical estimates could not account for patient-level differences in scene preparation, handover, or rendezvous processes. In addition, absolute total prehospital time may differ between traumatic and non-traumatic cases because of differences in on-scene care, patient stabilization, and destination selection. However, because the same estimation approach was applied to both HEMS and GEMS, this limitation is unlikely to have substantially biased the comparison between transport modalities. Finally, this study was conducted in a geographically isolated, medically underserved rural region; therefore, both the findings and our ‘absolute/relative’ necessity classification, which depends on local medical capacity and referral pathways, may have limited external generalizability and could be misclassified if applied to settings with different capabilities. In addition, this study focused on concordance between dispatch decisions and predefined necessity criteria and did not examine associations with clinical outcomes such as mortality or intensive care unit admission. Future studies incorporating patient-centered outcomes are needed to clarify the prognostic impact of dispatch appropriateness.

## Conclusions

In rural settings where local emergency care is available, clinical severity alone may be insufficient to determine the appropriateness of HEMS for non-traumatic emergencies. Our findings suggest that consideration of total prehospital time and geographic context may help refine dispatch decision-making and reduce misclassification of HEMS use in this specific setting.

## Supporting information

S1 Table List of Pre-Specified Diagnoses Evaluated for Relative Necessity of HEMS Dispatch. (DOCX)

S2 Table Emergency calls by area. (DOCX)

S3 Table Estimated total prehospital times by area and transport method. (DOCX)

S4 Table Final diagnosis of the patients judged as undertriage. (DOCX)

S5 Table Final diagnosis of the patients judged as overtriage. (DOCX)
