# Supplementary material for: Assessing the appropriateness of helicopter emergency medical services for non-traumatic emergencies in a medically underserved rural area, Japan
Source: PLoS One. 2026 Jul 9;21(7):e0353451. doi: 10.1371/journal.pone.0353451 (PMC13349173; doi:10.1371/journal.pone.0353451)
Supplement: S1 Table — (DOCX) [file pone.0353451.s001.docx]

**Supplementary Table 1. List of Pre-Specified Diagnoses Evaluated for Relative Necessity of HEMS Dispatch**

| **Neurological / Cardiovascular** | **Gastrointestinal / Abdominal** | **Infectious / Other** |
| --- | --- | --- |
| Cerebral infarction | Acute cholangitis | Necrotizing fasciitis |
| Intracerebral hemorrhage | Post-EMR colonic bleeding | Bacterial meningitis |
| Seizures and epilepsy | Sigmoid colon volvulus | Pyelonephritis |
| Subarachnoid hemorrhage | Sigmoid colon axial volvulus | Spontaneous bacterial peritonitis |
| Superior mesenteric artery dissection | Duodenal bleeding | Sepsis |
| Stanford type B aortic dissection | Duodenal perforation | Bacteremia |
| Heart failure | Duodenal varices | Anaphylactic shock |
| Myocardial infarction | Gastric and duodenal ulcers |  |
| Unstable angina | Anastomotic bleeding |  |
| Pulmonary embolism | Colonic perforation |  |
| Acute limb arterial occlusion | Small bowel perforation |  |
| Post-VF resuscitation | Acute abdominal syndrome |  |
| Ventricular tachycardia | Gastric ulcer perforation |  |
|  | Gastric varices |  |
|  | Esophageal varices |  |
|  | Strangulated inguinal hernia |  |
|  | Cholecystitis |  |
|  | Cholangitis |  |

HEMS, helicopter emergency medical service.
