# Supplementary material for: Assessing the appropriateness of helicopter emergency medical services for non-traumatic emergencies in a medically underserved rural area, Japan
Source: PLoS One. 2026 Jul 9;21(7):e0353451. doi: 10.1371/journal.pone.0353451 (PMC13349173; doi:10.1371/journal.pone.0353451)
Supplement: S2 Table — (DOCX) [file pone.0353451.s002.docx]

**Supplementary Table 2.　Emergency calls by area**

| Area | Primary request for HEMS dispatch  (n=63) | Secondary request for HEMS dispatch  (n=21) | Request for  GEMS dispatch  (n=2228) |
| --- | --- | --- | --- |
| A | 11 | 3 | 66 |
| B | 17 | 7 | 208 |
| C | 23 | 8 | 557 |
| D | 8 | 3 | 526 |
| E | 3 | 0 | 720 |
| F | 1 | 0 | 151 |

GEMS, ground emergency medical service; HEMS, helicopter emergency medical service.
