# Supplementary material for: Assessing the appropriateness of helicopter emergency medical services for non-traumatic emergencies in a medically underserved rural area, Japan
Source: PLoS One. 2026 Jul 9;21(7):e0353451. doi: 10.1371/journal.pone.0353451 (PMC13349173; doi:10.1371/journal.pone.0353451)
Supplement: S3 Table — (DOCX) [file pone.0353451.s003.docx]

**Supplementary Table 3. Estimated total prehospital times by area and transport method**

| Area | GEMS  Total prehospital time (min) | HEMS  Total prehospital time (min) |
| --- | --- | --- |
| A | 70 | 78 |
| B | 58 | 60 |
| C | 47 | 65 |
| D | 76 | 70 |
| E | 38 | 89 |
| F | 67 | 119 |

GEMS, ground emergency medical service; HEMS, helicopter emergency medical service.
