# Supplementary material for: Assessing the appropriateness of helicopter emergency medical services for non-traumatic emergencies in a medically underserved rural area, Japan
Source: PLoS One. 2026 Jul 9;21(7):e0353451. doi: 10.1371/journal.pone.0353451 (PMC13349173; doi:10.1371/journal.pone.0353451)
Supplement: S4 Table — (DOCX) [file pone.0353451.s004.docx]

**Supplementary Table 4. Final diagnosis of the patients judged as undertriage**

| Disease name |  | Number (%) of patients |
| --- | --- | --- |
| Specific diseases | Cerebral infraction | 60 (24.2) |
|  | Heart failure | 33 (13.3) |
|  | Intracerebral hemorrhage | 26 (10.5) |
|  | Acute pyelonephritis | 21 (8.5) |
|  | Bacterial pneumonia | 5 (2.0) |
|  | Dehydration | 3 (1.2) |
| Disease categories | Other diseases of the circulatory system other than cerebral infraction, intracerebral hemorrhage, and heart failure | 34 (13.7) |
|  | Others | 12(4.8) |
|  | Diseases of the digestive system | 36 (14.5) |
|  | Diseases of the nervous system | 20 (8.1) |
