# Supplementary material for: Assessing the appropriateness of helicopter emergency medical services for non-traumatic emergencies in a medically underserved rural area, Japan
Source: PLoS One. 2026 Jul 9;21(7):e0353451. doi: 10.1371/journal.pone.0353451 (PMC13349173; doi:10.1371/journal.pone.0353451)
Supplement: S5 Table — (DOCX) [file pone.0353451.s005.docx]

**Supplementary Table 5. Final diagnosis of the patients judged as overtriage**

| Disease name |  | Number (%) of patients |
| --- | --- | --- |
| Specific diseases | Cerebral infraction | 13 (23.2) |
|  | Intracerebral hemorrhage | 5 (8.9) |
|  | Bacterial pneumonia | 4 (7.1) |
|  | Acute pyelonephritis | 1(1.8) |
| Disease categories | Other diseases of the circulatory system other than cerebral infraction, intracerebral hemorrhage, and heart failure | 13 (23.2) |
|  | Diseases of the digestive system | 4 (7.1) |
|  | Other diseases of the respiratory system | 3 (5.4) |
|  | Other endocrine, nutritional or metabolic diseases | 3 (5.4) |
|  | Diseases of the nervous system | 2 (3.7) |
|  | Symptoms, signs or clinical findings, not elsewhere classified | 2 (3.7) |
|  | Other diseases of the genitourinary system | 2 (3.7) |
|  | Injury, poising or certain other consequences of external causes | 2 (3.7) |
|  | Neoplasms | 1 (1.8) |
|  | Diseases of the ear or mastoid process | 1 (1.8) |
