# Supplementary material for: Assessing the appropriateness of helicopter emergency medical services for non-traumatic emergencies in a medically underserved rural area, Japan
Source: PLoS One. 2026 Jul 9;21(7):e0353451. doi: 10.1371/journal.pone.0353451 (PMC13349173; doi:10.1371/journal.pone.0353451)
Supplement: Attachment — Submitted filename: Response_to_Reviewers.docx [file pone.0353451.s007.docx]

**Response to Editor and Reviewers**

We would like to thank the editor and both reviewers for their thoughtful and constructive comments, which have significantly improved the quality of our manuscript. We have carefully addressed all suggestions and revised the manuscript accordingly. All major changes are described below in a point-by-point format, and all revisions have been marked using the track changes function in the manuscript.

Below, we provide a point-by-point response to each comment.

**Reviewer #1**

Thank you very much for your positive and helpful comments. We recognize that undertriage remains an important issue in HEMS dispatch. We also agree that overtriage should be interpreted carefully, because some degree of overtriage may be acceptable to reduce clinically important undertriage.

**Reviewer #2**

1. **Line 46: Methods. The line reads: ‘HEMS necessity was classified as “absolute” (treatment unavailable at the locally) or…’ What is meant by locally? Is it local hospital. Please elaborate.**

Response: Thank you for this helpful comment. The phrase “at the locally” was unclear and grammatically incorrect. By “locally,” we meant local core hospitals in the study region. Specifically, “absolute necessity” referred to cases requiring treatments or specialist capabilities that were unavailable at local core hospitals and therefore required transfer to tertiary emergency facilities. We have revised the Abstract to make this definition consistent with the Methods section.

2. **The introduction is satisfactory and adequately written, establishing the context and rationale for the study. It provides a clear framework for the research questions and objectives.Minor remarks: Line 78: ‘ …and others nothing delays in treatment for non-trauma cases transported by HEMS…’ What is meant by ‘nothing’? Should this be ‘noting’?**

Response: Thank you for pointing this out. “Nothing” was a typographical error and should have been “noting.” We have corrected the sentence to indicate that some studies have noted delays in treatment among non-trauma cases transported by HEMS.

3. **Settings needs to describe in clearer terms, which level the hospitals in the region are. ‘Main local core hospital’ is not a term familiar to me. Please be more specific on which competencies are available. Are all hospitals stroke-centers with acute thrombolytic capabilities? Are cat labs available? Or have these functions been centralized.**

Response: Thank you for this helpful comment. The term “main local core hospital” was not sufficiently clear for international readers. We have revised the Study Design and Setting section to describe the hospital-level capabilities in the study region more specifically. In brief, the local core hospital provides 24-hour emergency care and inpatient management for common non-traumatic emergencies, including stroke, acute coronary syndrome, heart failure, pneumonia, and sepsis. Acute thrombolytic therapy and emergency coronary catheterization can be provided at the local core hospital, although selected advanced cases require transfer to tertiary emergency medical centers in Kochi City. We also clarified that advanced tertiary-level interventions, including surgical management of Stanford type A aortic dissection, emergency revascularization for acute myocardial infarction requiring advanced management of three-vessel disease, and catheter-based intervention for pulmonary embolism, are centralized in tertiary emergency medical centers in Kochi City.

4. **Next, please describe which ambulance levels are available. It is written, that a physician is in the HEMS. Is there physician staffed vehicles as well? Or are ambulances always EMT/paramedic?**

Response: Thank you for this helpful comment. We have revised the Study Design and Setting section to clarify the levels of ambulance services available in the study region. Ground ambulances are staffed by emergency medical technicians, including paramedics, and do not carry physicians. We also clarified that physician-staffed ground vehicles are not routinely available in the study region.

5. **Regarding variables, please describe in further detail which variables are available in the dispatch data on GEMS transport. The definition of transport-time as time from call to arrival to hospital is somewhat controversial. Usually, prehospital time is divided into response-time (call to arrival), on-scene-time (from arrival to departure) and Transport-time (time from departure to arrival to hospital). Consider using the more traditional transport-time or argue why the entire prehospital time is used.**

Response: Thank you for this important comment. We have revised the Variables section to describe the variables available in the GEMS dispatch data in greater detail. We also clarified our time definition. In the original manuscript, we used the term “transport time” to refer to the total time from the initial emergency call to arrival at the receiving hospital. However, as the reviewer correctly noted, this differs from the conventional definition of transport time, which usually refers to the interval from scene departure to hospital arrival. Our aim was to evaluate the appropriateness of the initial dispatch decision, that is, whether HEMS should have been requested at the time of the emergency call. Therefore, we considered the total time from call to hospital arrival to be more relevant than the post-departure transport interval alone. To avoid confusion, we have revised the term “transport time” to “total prehospital time” throughout the manuscript when referring to this interval.

6. **In the definition of Relative necessity, part of the definition is ‘The patient was considered clinically unstable upon arrival’. Which arrival are we discussing here? Is it EMS arrival to scene, or patient arrival to hospital? If arrival to hospital, this definition is flawed by the prehospital treatment, depending on level of care delivered by EMS-personnel. Please elaborate on this.**

Response: Thank you for this helpful comment. The phrase “upon arrival” was ambiguous, and we have revised it to “on arrival at the receiving hospital.” We acknowledge that vital signs at hospital arrival may be affected by prehospital treatment. However, our aim was not to reproduce real-time dispatch decision-making based on the initial field assessment, but to retrospectively assess whether HEMS use was justified based on final diagnosis, physiological status on arrival at the receiving hospital, total prehospital time, and regional access to definitive care. Therefore, hospital-arrival vital signs were used as markers of persistent hemodynamic or respiratory instability after prehospital care. We have clarified this point in the Methods section.

7. **It is not absolutely clear how groups are compared. It is described, how ‘To evaluate relative necessity, we compared observed transport times to estimated GEMS only travel times using historical trauma data from each region. Estimations were stratified by departure area and receiving facility.’. How is transports with GEMS only simulated? Would this be by the theoretically calculated HEMS time? And would this be by true transport-time, or total prehospital time for HEMS? If the latter, this would skew the estimates, as at least in my EMS, the GEMS prepare the patients, and the HEMS would transport the patient to the specialised facilities. In general, the historic transport times are opaque. Is this an average for all transports from the area, or is it more specific?**

Response: Thank you for this important comment. We have revised the Methods section to clarify how the time comparison was performed. We did not simulate patient-level GEMS-only or HEMS transport times. Instead, we compared mean historical total prehospital times for HEMS and GEMS using trauma transport data from the same region. Mean values were calculated separately for each combination of departure area and receiving facility. HEMS was considered to provide a time advantage when the mean historical total prehospital time for HEMS was shorter than that for GEMS within the same departure-area and receiving-facility combination. We also clarified that, in actual HEMS missions, ground EMS may assess the patient, provide initial care, prepare the patient for transport, or transport the patient to a rendezvous point before handover to HEMS. These operational processes were not individually modeled in our area-level historical estimates. We have clarified this approach in the Methods section and added this issue as a limitation.

8. **The results section starts with 23,327 participants, but population flowcharts starts with 3,201 participants Sometimes only percentages are given, and sometimes both absolute numbers and percentages are given. Please always state absolute numbers as well as percentages.**

Response: Thank you for this careful comment. To avoid confusion, we revised the population flowchart to begin with all 23,327 ambulance requests recorded during the study period. The flowchart now shows that 3,201 cases met the initial inclusion criteria, followed by the exclusion of 889 cases, resulting in 2,312 patients included in the final analysis. We also revised the Results section to ensure consistency with the flowchart and to report both absolute numbers and percentages throughout the manuscript.

9. **In line 225 it is stated ‘However, only 253 of these patients fulfilled the full criteria for relative necessity, which required both an eligible diagnosis and a shorter estimated transport time via HEMS compared to GEMS from their respective locations.’. Is this transport time to the selected hospital the patient were historically transported to? Please make sure the methods reflects how this is measured.**

Response: Thank you for this helpful comment. In this study, the time comparison was based on the receiving hospital to which each patient was actually transported. We did not assume transport to a theoretical or different destination. For each patient, we identified the corresponding departure-area and receiving-hospital combination and compared the mean historical total prehospital times for HEMS and GEMS within that same combination. These mean values were derived from observed historical trauma transport data. We have revised the Methods section to clarify that the destination hospital used for the comparison was the hospital to which the patient was actually transported.

10. **The more I read on transport times, the more I am worried about the calculation. Do your EMS ever fly rendezvous missions, where the GEMS starts transports and meet the HEMS somewhere and the patient are loaded onto the HEMS for final leg of the transport? Looking at supplementary 3, these times are bizarre. How do you calculate these mission times? It really needs a breakdown of the individual time segments of prehospital times**

Response: Thank you for this important comment. We understand the reviewer’s concern regarding the interpretation of the mission times. We have revised the title and related descriptions of Supplementary Table 3 from “transport time” to “total prehospital time,” defined as the interval from the initial emergency call to arrival at the receiving hospital.

In our EMS system, ground EMS may assess the patient, provide initial care, prepare the patient for transport, or transport the patient to a rendezvous point before handover to HEMS. Therefore, HEMS missions may include varying degrees of GEMS involvement. However, the objective of this study was not to isolate helicopter flight time or to quantify the extent of GEMS involvement during each mission. Rather, our aim was to evaluate whether the decision to use HEMS was justified from a system-level perspective, based on whether HEMS use was associated with a shorter total prehospital time to the actual receiving hospital. We have clarified this rationale in the Methods section and added the inability to model patient-level GEMS-HEMS operational processes as a limitation.
