# Supplementary material for: Assessing the appropriateness of helicopter emergency medical services for non-traumatic emergencies in a medically underserved rural area, Japan
Source: PLoS One. 2026 Jul 9;21(7):e0353451. doi: 10.1371/journal.pone.0353451 (PMC13349173; doi:10.1371/journal.pone.0353451)
Supplement: Peer review history

# Peer review: Decision Letter 0

**DOI:** 10.1371/journal.pone.0353451.r001

**Authors:** Pawan Acharya

11 May 2026

PONE-D-26-10437

Assessing the appropriateness of helicopter emergency medical services for non-traumatic emergencies in a medically underserved rural area, Japan

PLOS One

Dear Dr. Sada,

Thank you for submitting your manuscript to PLOS ONE. After careful consideration, we feel that it has merit but does not fully meet PLOS ONE’s publication criteria as it currently stands. Therefore, we invite you to submit a revised version of the manuscript that addresses the points raised during the review process.

==============================

Thank you for submitting your manuscript examining overtriage and undertriage of helicopter emergency medical services (HEMS) for nontraumatic emergencies. The reviewers found the topic to be clinically important and appreciated the inclusion of both HEMS and GEMS missions in the analysis. While one reviewer considered the manuscript acceptable in its current form, another reviewer raised several important concerns regarding clarity of the EMS system description, terminology, and particularly the methodology used to define and compare transport times and relative necessity. After consideration of the reviews, I believe the manuscript would benefit from revision to improve methodological transparency and interpretation of the findings. Please address the reviewer comments carefully and provide detailed responses to each point raised.

**Comments from reviewer 1:**

Thank you for the opportunity to review this important work. Overall, this is a well-designed study with rigorous methods and a great first step. Under-triage remains an issue. I don't necessarily see over-triage as a problem, but physiological indicators could go a long way to address both. This is named as a limitation.

**Comments from reviewer 2:**

This manuscript presents an interesting study examining overtriage and undertriage of HEMS for non-traumatic missions in a Japanese region.

I would like to commend the study for addressing this important topic, as this study differs from other studies by analysing both GEMS and HEMS missions to investigate the subject.

I have reviewed the manuscript carefully and to the best of my ability. If I have misunderstood any aspects, I apologize; however, if such misunderstandings arise, this may also point to areas where the manuscript could be clarified for readers.

Abstract

The abstract summarises the study satisfactorily.

Minor remarks:

Line 46: Methods. The line reads: ‘HEMS necessity was classified as “absolute” (treatment

unavailable at the locally) or…’ What is meant by locally? Is it local hospital. Please elaborate.

Introduction

The introduction is satisfactory and adequately written, establishing the context and rationale for the study. It provides a clear framework for the research questions and objectives.

Minor remarks: Line 78: ‘ …and others nothing delays in treatment for non-trauma cases transported by HEMS…’ What is meant by ‘nothing’? Should this be ‘noting’?

Methods:

Settings needs to describe in clearer terms, which level the hospitals in the region are. ‘Main local core hospital’ is not a term familiar to me. Please be more specific on which competencies are available. Are all hospitals stroke-centers with acute thrombolytic capabilities? Are cat labs available? Or have these functions been centralized.

Next, please describe which ambulance levels are available. It is written, that a physician is in the HEMS. Is there physician staffed vehicles as well? Or are ambulances always EMT/paramedic?

Regarding variables, please describe in further detail which variables are available in the dispatch data on GEMS transport.

The definition of transport-time as time from call to arrival to hospital is somewhat controversial. Usually, prehospital time is divided into response-time (call to arrival), on-scene-time (from arrival to departure) and Transport-time (time from departure to arrival to hospital). Consider using the more traditional transport-time or argue why the entire prehospital time is used.

In the definition of Relative necessity, part of the definition is ‘The patient was considered clinically unstable upon arrival’. Which arrival are we discussing here? Is it EMS arrival to scene, or patient arrival to hospital? If arrival to hospital, this definition is flawed by the prehospital treatment, depending on level of care delivered by EMS-personnel. Please elaborate on this.

It is not absolutely clear how groups are compared. It is described, how ‘To evaluate relative necessity, we compared observed transport times to estimated GEMS only travel times using historical trauma data from each region. Estimations were stratified by departure area and receiving facility.’. How is transports with GEMS only simulated? Would this be by the theoretically calculated HEMS time? And would this be by true transport-time, or total prehospital time for HEMS? If the latter, this would skew the estimates, as at least in my EMS, the GEMS prepare the patients, and the HEMS would transport the patient to the specialised facilities. In general, the historic transport times are opaque. Is this an average for all transports from the area, or is it more specific?

Results:

The results section starts with 23,327 participants, but population flowcharts starts with 3,201 participants

Sometimes only percentages are given, and sometimes both absolute numbers and percentages are given. Please always state absolute numbers as well as percentages.

In line 225 it is stated ‘However, only 253 of these patients fulfilled the full criteria for relative necessity, which required both an eligible diagnosis and a shorter estimated transport time via HEMS compared to GEMS from their respective locations.’. Is this transport time to the selected hospital the patient were historically transported to? Please make sure the methods reflects how this is measured.

The more I read on transport times, the more I am worried about the calculation. Do your EMS ever fly rendezvous missions, where the GEMS starts transports and meet the HEMS somewhere and the patient are loaded onto the HEMS for final leg of the transport? Looking at supplementary 3, these times are bizarre. How do you calculate these mission times? It really needs a breakdown of the individual time segments of prehospital times

Discussion

Discussion is adequate.

Conclusion is supported by the presented data. However, as indicated, I am not entirely convinced the data presented are brought to the best use. It appears to dismiss the cooperation of HEMS and GEMS, where HEMS fly time to mission is being used by GEMS to prep the patient for transport.

==============================

Please submit your revised manuscript by Jun 25 2026 11:59PM. If you will need more time than this to complete your revisions, please reply to this message or contact the journal office at plosone@plos.org. When you're ready to submit your revision, log on to https://www.editorialmanager.com/pone/ and select the 'Submissions Needing Revision' folder to locate your manuscript file.

Please include the following items when submitting your revised manuscript:

- A letter that responds to each point raised by the academic editor and reviewer(s). You should upload this letter as a separate file labeled 'Response to Reviewers'.
- A marked-up copy of your manuscript that highlights changes made to the original version. You should upload this as a separate file labeled 'Revised Manuscript with Track Changes'.
- An unmarked version of your revised paper without tracked changes. You should upload this as a separate file labeled 'Manuscript'.

If you would like to make changes to your financial disclosure, please include your updated statement in your cover letter. Guidelines for resubmitting your figure files are available below the reviewer comments at the end of this letter.

If applicable, we recommend that you deposit your laboratory protocols in protocols.io to enhance the reproducibility of your results. Protocols.io assigns your protocol its own identifier (DOI) so that it can be cited independently in the future. For instructions see: https://journals.plos.org/plosone/s/submission-guidelines#loc-laboratory-protocols. Additionally, PLOS ONE offers an option for publishing peer-reviewed Lab Protocol articles, which describe protocols hosted on protocols.io. Read more information on sharing protocols at https://plos.org/protocols?utm_medium=editorial-email&utm_source=authorletters&utm_campaign=protocols.

As the corresponding author, your ORCID iD is verified in the submission system and will appear in the published article. PLOS supports the use of ORCID, and we encourage all coauthors to register for an ORCID iD and use it as well. Please encourage your coauthors to verify their ORCID iD within the submission system before final acceptance, as unverified ORCID iDs will not appear in the published article. *Only*  the individual author can complete the verification step; PLOS staff *cannot*  verify ORCID iDs on behalf of authors.

We look forward to receiving your revised manuscript.

Kind regards,

Pawan Acharya, PhD, MSPH

Academic Editor

PLOS One

Journal Requirements:

When submitting your revision, we need you to address these additional requirements.

1. Please ensure that your manuscript meets PLOS ONE's style requirements, including those for file naming. The PLOS ONE style templates can be found at

https://journals.plos.org/plosone/s/file?id=wjVg/PLOSOne_formatting_sample_main_body.pdf and

https://journals.plos.org/plosone/s/file?id=ba62/PLOSOne_formatting_sample_title_authors_affiliations.pdf.

2. We note that you have indicated that there are restrictions to data sharing for this study. For studies involving human research participant data or other sensitive data, we encourage authors to share de-identified or anonymized data. However, when data cannot be publicly shared for ethical reasons, we allow authors to make their data sets available upon request. For information on unacceptable data access restrictions, please see http://journals.plos.org/plosone/s/data-availability#loc-unacceptable-data-access-restrictions.

Before we proceed with your manuscript, please address the following prompts:

a) If there are ethical or legal restrictions on sharing a de-identified data set, please explain them in detail (e.g., data contain potentially identifying or sensitive patient information, data are owned by a third-party organization, etc.) and who has imposed them (e.g., a Research Ethics Committee or Institutional Review Board, etc.). Please also provide contact information for a data access committee, ethics committee, or other institutional body to which data requests may be sent.

b) If there are no restrictions, please upload the minimal anonymized data set necessary to replicate your study findings to a stable, public repository and provide us with the relevant URLs, DOIs, or accession numbers. Please see http://www.bmj.com/content/340/bmj.c181.long for guidelines on how to de-identify and prepare clinical data for publication. For a list of recommended repositories, please see https://journals.plos.org/plosone/s/recommended-repositories. You also have the option of uploading the data as Supporting Information files, but we would recommend depositing data directly to a data repository if possible.

Please update your Data Availability statement in the submission form accordingly.

3. In the online submission form you indicate that your data is not available for proprietary reasons and have provided a contact point for accessing this data. Please note that your current contact point is a co-author on this manuscript. According to our Data Policy, the contact point must not be an author on the manuscript and must be an institutional contact, ideally not an individual. Please revise your data statement to a non-author institutional point of contact, such as a data access or ethics committee, and send this to us via return email. Please also include contact information for the third party organization, and please include the full citation of where the data can be found.

4.We note that Figures 1 in your submission contain [map/satellite] images which may be copyrighted. All PLOS content is published under the Creative Commons Attribution License (CC BY 4.0), which means that the manuscript, images, and Supporting Information files will be freely available online, and any third party is permitted to access, download, copy, distribute, and use these materials in any way, even commercially, with proper attribution. For these reasons, we cannot publish previously copyrighted maps or satellite images created using proprietary data, such as Google software (Google Maps, Street View, and Earth). For more information, see our copyright guidelines: http://journals.plos.org/plosone/s/licenses-and-copyright.

We require you to either (1) present written permission from the copyright holder to publish these figures specifically under the CC BY 4.0 license, or (2) remove the figures from your submission:

a. You may seek permission from the original copyright holder of Figures 1 to publish the content specifically under the CC BY 4.0 license.

We recommend that you contact the original copyright holder with the Content Permission Form (http://journals.plos.org/plosone/s/file?id=7c09/content-permission-form.pdf) and the following text:

“I request permission for the open-access journal PLOS ONE to publish XXX under the Creative Commons Attribution License (CCAL) CC BY 4.0 (http://creativecommons.org/licenses/by/4.0/). Please be aware that this license allows unrestricted use and distribution, even commercially, by third parties. Please reply and provide explicit written permission to publish XXX under a CC BY license and complete the attached form.”

Please upload the completed Content Permission Form or other proof of granted permissions as an "Other" file with your submission.

In the figure caption of the copyrighted figure, please include the following text: “Reprinted from [ref] under a CC BY license, with permission from [name of publisher], original copyright [original copyright year].”

b. If you are unable to obtain permission from the original copyright holder to publish these figures under the CC BY 4.0 license or if the copyright holder’s requirements are incompatible with the CC BY 4.0 license, please either i) remove the figure or ii) supply a replacement figure that complies with the CC BY 4.0 license. Please check copyright information on all replacement figures and update the figure caption with source information. If applicable, please specify in the figure caption text when a figure is similar but not identical to the original image and is therefore for illustrative purposes only.

The following resources for replacing copyrighted map figures may be helpful:

USGS National Map Viewer (public domain): http://viewer.nationalmap.gov/viewer/

The Gateway to Astronaut Photography of Earth (public domain): http://eol.jsc.nasa.gov/sseop/clickmap/

Maps at the CIA (public domain): https://www.cia.gov/library/publications/the-world-factbook/index.html and https://www.cia.gov/library/publications/cia-maps-publications/index.html

NASA Earth Observatory (public domain): http://earthobservatory.nasa.gov/

Landsat: http://landsat.visibleearth.nasa.gov/

USGS EROS (Earth Resources Observatory and Science (EROS) Center) (public domain): http://eros.usgs.gov/#

Natural Earth (public domain): http://www.naturalearthdata.com/

5. If the reviewer comments include a recommendation to cite specific previously published works, please review and evaluate these publications to determine whether they are relevant and should be cited. There is no requirement to cite these works unless the editor has indicated otherwise.

Additional Editor Comments (if provided):

Thank you for submitting your manuscript examining overtriage and undertriage of helicopter emergency medical services (HEMS) for nontraumatic emergencies. The reviewers found the topic to be clinically important and appreciated the inclusion of both HEMS and GEMS missions in the analysis. While one reviewer considered the manuscript acceptable in its current form, another reviewer raised several important concerns regarding clarity of the EMS system description, terminology, and particularly the methodology used to define and compare transport times and relative necessity. After consideration of the reviews, I believe the manuscript would benefit from revision to improve methodological transparency and interpretation of the findings. Please address the reviewer comments carefully and provide detailed responses to each point raised.

[Note: HTML markup is below. Please do not edit.]

Reviewers' comments:

Reviewer's Responses to Questions

**Comments to the Author**

1. Is the manuscript technically sound, and do the data support the conclusions?

The manuscript must describe a technically sound piece of scientific research with data that supports the conclusions. Experiments must have been conducted rigorously, with appropriate controls, replication, and sample sizes. The conclusions must be drawn appropriately based on the data presented.

Reviewer #1: Yes

Reviewer #2: Partly

**********

2. Has the statistical analysis been performed appropriately and rigorously?

Reviewer #1: Yes

Reviewer #2: Yes

**********

3. Have the authors made all data underlying the findings in their manuscript fully available?

The PLOS Data policy requires authors to make all data underlying the findings described in their manuscript fully available without restriction, with rare exception (please refer to the Data Availability Statement in the manuscript PDF file). The data should be provided as part of the manuscript or its supporting information, or deposited to a public repository. For example, in addition to summary statistics, the data points behind means, medians and variance measures should be available. If there are restrictions on publicly sharing data—e.g. participant privacy or use of data from a third party—those must be specified.

Reviewer #1: Yes

Reviewer #2: No

**********

4. Is the manuscript presented in an intelligible fashion and written in standard English?

PLOS ONE does not copyedit accepted manuscripts, so the language in submitted articles must be clear, correct, and unambiguous. Any typographical or grammatical errors should be corrected at revision, so please note any specific errors here.

Reviewer #1: Yes

Reviewer #2: Yes

**********

5. Review Comments to the Author

Please use the space provided to explain your answers to the questions above. You may also include additional comments for the author, including concerns about dual publication, research ethics, or publication ethics. (Please upload your review as an attachment if it exceeds 20,000 characters)

Reviewer #1: Thank you for the opportunity to review this important work. Overall, this is a well-designed study with rigorous methods and a great first step. Under-triage remains an issue. I don't necessarily see over-triage as a problem, but physiological indicators could go a long way to address both. This is named as a limitation.

Reviewer #2: Review Hems Japan

This manuscript presents an interesting study examining overtriage and undertriage of HEMS for non-traumatic missions in a Japanese region.

I would like to commend the study for addressing this important topic, as this study differs from other studies by analysing both GEMS and HEMS missions to investigate the subject.

I have reviewed the manuscript carefully and to the best of my ability. If I have misunderstood any aspects, I apologize; however, if such misunderstandings arise, this may also point to areas where the manuscript could be clarified for readers.

Abstract

The abstract summarises the study satisfactorily.

Minor remarks:

Line 46: Methods. The line reads: ‘HEMS necessity was classified as “absolute” (treatment

unavailable at the locally) or…’ What is meant by locally? Is it local hospital. Please elaborate.

Introduction

The introduction is satisfactory and adequately written, establishing the context and rationale for the study. It provides a clear framework for the research questions and objectives.

Minor remarks: Line 78: ‘ …and others nothing delays in treatment for non-trauma cases transported by HEMS…’ What is meant by ‘nothing’? Should this be ‘noting’?

Methods:

Settings needs to describe in clearer terms, which level the hospitals in the region are. ‘Main local core hospital’ is not a term familiar to me. Please be more specific on which competencies are available. Are all hospitals stroke-centres with acute thrombolytic capabilities? Are cat labs available? Or have these functions been centralised.

Next, please describe which ambulance levels are available. It is written, that a physician is in the HEMS. Is there physician staffed vehicles as well? Or are ambulances always EMT/paramedic?

Regarding variables, please describe in further detail which variables are available in the dispatch data on GEMS transport.

The definition of transport-time as time from call to arrival to hospital is somewhat controversial. Usually, prehospital time is divided into response-time (call to arrival), on-scene-time (from arrival to departure) and Transport-time (time from departure to arrival to hospital). Consider using the more traditional transport-time or argue why the entire prehospital time is used.

In the definition of Relative necessity, part of the definition is ‘The patient was considered clinically unstable upon arrival’. Which arrival are we discussing here? Is it EMS arrival to scene, or patient arrival to hospital? If arrival to hospital, this definition is flawed by the prehospital treatment, depending on level of care delivered by EMS-personnel. Please elaborate on this.

It is not absolutely clear how groups are compared. It is described, how ‘To evaluate relative necessity, we compared observed transport times to estimated GEMS only travel times using historical trauma data from each region. Estimations were stratified by departure area and receiving facility.’. How is transports with GEMS only simulated? Would this be by the theoretically calculated HEMS time? And would this be by true transport-time, or total prehospital time for HEMS? If the latter, this would skew the estimates, as at least in my EMS, the GEMS prepare the patients, and the HEMS would transport the patient to the specialised facilities. In general, the historic transport times are opaque. Is this an average for all transports from the area, or is it more specific?

Results:

The results section starts with 23,327 participants, but population flowcharts starts with 3,201 participants

Sometimes only percentages are given, and sometimes both absolute numbers and percentages are given. Please always state absolute numbers as well as percentages.

In line 225 it is stated ‘However, only 253 of these patients fulfilled the full criteria for relative necessity, which required both an eligible diagnosis and a shorter estimated transport time via HEMS compared to GEMS from their respective locations.’. Is this transport time to the selected hospital the patient were historically transported to? Please make sure the methods reflects how this is measured.

The more I read on transporttimes, the more I am worried about the calculation. Do your EMS ever fly rendez vous missions, where the GEMS starts transports and meet the HEMS somewhere and the patient are loaded onto the HEMS for final leg of the transport? Looking at supplementary 3, these times are bizarre. How do you calculate these missiontimes? It really needs a breakdown of the individual timesegments of prehospital times

Discussion

Discussion is adequate.

Conclusion is supported by the presented data. However, as indicated, I am not entirely convinced the data presented are brought to the best use. It appears to dismiss the cooperation of HEMS and GEMS, where HEMS fly time to mission is being used by GEMS to prep the patient for transport.

**********

6. PLOS authors have the option to publish the peer review history of their article (what does this mean?). If published, this will include your full peer review and any attached files.

If you choose “no”, your identity will remain anonymous but your review may still be made public.

**Do you want your identity to be public for this peer review?**  For information about this choice, including consent withdrawal, please see our Privacy Policy.

Reviewer #1: No

Reviewer #2: No

**********

[NOTE: If reviewer comments were submitted as an attachment file, they will be attached to this email and accessible via the submission site. Please log into your account, locate the manuscript record, and check for the action link "View Attachments". If this link does not appear, there are no attachment files.]

To ensure your figures meet our technical requirements, please review our figure guidelines: https://journals.plos.org/plosone/s/figures

You may also use PLOS’s free figure tool, NAAS, to help you prepare publication quality figures: https://journals.plos.org/plosone/s/figures#loc-tools-for-figure-preparation.

NAAS will assess whether your figures meet our technical requirements by comparing each figure against our figure specifications.

# Author comment: Author response to Decision Letter 1

**DOI:** 10.1371/journal.pone.0353451.r002

20 May 2026

Response to Editor and Reviewers

We would like to thank the editor and both reviewers for their thoughtful and constructive comments, which have significantly improved the quality of our manuscript. We have carefully addressed all suggestions and revised the manuscript accordingly. All major changes are described below in a point-by-point format, and all revisions have been marked using the track changes function in the manuscript.

Below, we provide a point-by-point response to each comment.

Reviewer #1

Thank you very much for your positive and helpful comments. We recognize that undertriage remains an important issue in HEMS dispatch. We also agree that overtriage should be interpreted carefully, because some degree of overtriage may be acceptable to reduce clinically important undertriage.

Reviewer #2

1. Line 46: Methods. The line reads: ‘HEMS necessity was classified as “absolute” (treatment unavailable at the locally) or…’ What is meant by locally? Is it local hospital. Please elaborate.

Response: Thank you for this helpful comment. The phrase “at the locally” was unclear and grammatically incorrect. By “locally,” we meant local core hospitals in the study region. Specifically, “absolute necessity” referred to cases requiring treatments or specialist capabilities that were unavailable at local core hospitals and therefore required transfer to tertiary emergency facilities. We have revised the Abstract to make this definition consistent with the Methods section.

2. The introduction is satisfactory and adequately written, establishing the context and rationale for the study. It provides a clear framework for the research questions and objectives.Minor remarks: Line 78: ‘ …and others nothing delays in treatment for non-trauma cases transported by HEMS…’ What is meant by ‘nothing’? Should this be ‘noting’?

Response: Thank you for pointing this out. “Nothing” was a typographical error and should have been “noting.” We have corrected the sentence to indicate that some studies have noted delays in treatment among non-trauma cases transported by HEMS.

3. Settings needs to describe in clearer terms, which level the hospitals in the region are. ‘Main local core hospital’ is not a term familiar to me. Please be more specific on which competencies are available. Are all hospitals stroke-centers with acute thrombolytic capabilities? Are cat labs available? Or have these functions been centralized.

Response: Thank you for this helpful comment. The term “main local core hospital” was not sufficiently clear for international readers. We have revised the Study Design and Setting section to describe the hospital-level capabilities in the study region more specifically. In brief, the local core hospital provides 24-hour emergency care and inpatient management for common non-traumatic emergencies, including stroke, acute coronary syndrome, heart failure, pneumonia, and sepsis. Acute thrombolytic therapy and emergency coronary catheterization can be provided at the local core hospital, although selected advanced cases require transfer to tertiary emergency medical centers in Kochi City. We also clarified that advanced tertiary-level interventions, including surgical management of Stanford type A aortic dissection, emergency revascularization for acute myocardial infarction requiring advanced management of three-vessel disease, and catheter-based intervention for pulmonary embolism, are centralized in tertiary emergency medical centers in Kochi City.

4. Next, please describe which ambulance levels are available. It is written, that a physician is in the HEMS. Is there physician staffed vehicles as well? Or are ambulances always EMT/paramedic?

Response: Thank you for this helpful comment. We have revised the Study Design and Setting section to clarify the levels of ambulance services available in the study region. Ground ambulances are staffed by emergency medical technicians, including paramedics, and do not carry physicians. We also clarified that physician-staffed ground vehicles are not routinely available in the study region.

5. Regarding variables, please describe in further detail which variables are available in the dispatch data on GEMS transport. The definition of transport-time as time from call to arrival to hospital is somewhat controversial. Usually, prehospital time is divided into response-time (call to arrival), on-scene-time (from arrival to departure) and Transport-time (time from departure to arrival to hospital). Consider using the more traditional transport-time or argue why the entire prehospital time is used.

Response: Thank you for this important comment. We have revised the Variables section to describe the variables available in the GEMS dispatch data in greater detail. We also clarified our time definition. In the original manuscript, we used the term “transport time” to refer to the total time from the initial emergency call to arrival at the receiving hospital. However, as the reviewer correctly noted, this differs from the conventional definition of transport time, which usually refers to the interval from scene departure to hospital arrival. Our aim was to evaluate the appropriateness of the initial dispatch decision, that is, whether HEMS should have been requested at the time of the emergency call. Therefore, we considered the total time from call to hospital arrival to be more relevant than the post-departure transport interval alone. To avoid confusion, we have revised the term “transport time” to “total prehospital time” throughout the manuscript when referring to this interval.

6. In the definition of Relative necessity, part of the definition is ‘The patient was considered clinically unstable upon arrival’. Which arrival are we discussing here? Is it EMS arrival to scene, or patient arrival to hospital? If arrival to hospital, this definition is flawed by the prehospital treatment, depending on level of care delivered by EMS-personnel. Please elaborate on this.

Response: Thank you for this helpful comment. The phrase “upon arrival” was ambiguous, and we have revised it to “on arrival at the receiving hospital.” We acknowledge that vital signs at hospital arrival may be affected by prehospital treatment. However, our aim was not to reproduce real-time dispatch decision-making based on the initial field assessment, but to retrospectively assess whether HEMS use was justified based on final diagnosis, physiological status on arrival at the receiving hospital, total prehospital time, and regional access to definitive care. Therefore, hospital-arrival vital signs were used as markers of persistent hemodynamic or respiratory instability after prehospital care. We have clarified this point in the Methods section.

7. It is not absolutely clear how groups are compared. It is described, how ‘To evaluate relative necessity, we compared observed transport times to estimated GEMS only travel times using historical trauma data from each region. Estimations were stratified by departure area and receiving facility.’. How is transports with GEMS only simulated? Would this be by the theoretically calculated HEMS time? And would this be by true transport-time, or total prehospital time for HEMS? If the latter, this would skew the estimates, as at least in my EMS, the GEMS prepare the patients, and the HEMS would transport the patient to the specialised facilities. In general, the historic transport times are opaque. Is this an average for all transports from the area, or is it more specific?

Response: Thank you for this important comment. We have revised the Methods section to clarify how the time comparison was performed. We did not simulate patient-level GEMS-only or HEMS transport times. Instead, we compared mean historical total prehospital times for HEMS and GEMS using trauma transport data from the same region. Mean values were calculated separately for each combination of departure area and receiving facility. HEMS was considered to provide a time advantage when the mean historical total prehospital time for HEMS was shorter than that for GEMS within the same departure-area and receiving-facility combination. We also clarified that, in actual HEMS missions, ground EMS may assess the patient, provide initial care, prepare the patient for transport, or transport the patient to a rendezvous point before handover to HEMS. These operational processes were not individually modeled in our area-level historical estimates. We have clarified this approach in the Methods section and added this issue as a limitation.

8. The results section starts with 23,327 participants, but population flowcharts starts with 3,201 participants Sometimes only percentages are given, and sometimes both absolute numbers and percentages are given. Please always state absolute numbers as well as percentages.

Response: Thank you for this careful comment. To avoid confusion, we revised the population flowchart to begin with all 23,327 ambulance requests recorded during the study period. The flowchart now shows that 3,201 cases met the initial inclusion criteria, followed by the exclusion of 889 cases, resulting in 2,312 patients included in the final analysis. We also revised the Results section to ensure consistency with the flowchart and to report both absolute numbers and percentages throughout the manuscript.

9. In line 225 it is stated ‘However, only 253 of these patients fulfilled the full criteria for relative necessity, which required both an eligible diagnosis and a shorter estimated transport time via HEMS compared to GEMS from their respective locations.’. Is this transport time to the selected hospital the patient were historically transported to? Please make sure the methods reflects how this is measured.

Response: Thank you for this helpful comment. In this study, the time comparison was based on the receiving hospital to which each patient was actually transported. We did not assume transport to a theoretical or different destination. For each patient, we identified the corresponding departure-area and receiving-hospital combination and compared the mean historical total prehospital times for HEMS and GEMS within that same combination. These mean values were derived from observed historical trauma transport data. We have revised the Methods section to clarify that the destination hospital used for the comparison was the hospital to which the patient was actually transported.

10. The more I read on transport times, the more I am worried about the calculation. Do your EMS ever fly rendezvous missions, where the GEMS starts transports and meet the HEMS somewhere and the patient are loaded onto the HEMS for final leg of the transport? Looking at supplementary 3, these times are bizarre. How do you calculate these mission times? It really needs a breakdown of the individual time segments of prehospital times

Response: Thank you for this important comment. We understand the reviewer’s concern regarding the interpretation of the mission times. We have revised the title and related descriptions of Supplementary Table 3 from “transport time” to “total prehospital time,” defined as the interval from the initial emergency call to arrival at the receiving hospital.

In our EMS system, ground EMS may assess the patient, provide initial care, prepare the patient for transport, or transport the patient to a rendezvous point before handover to HEMS. Therefore, HEMS missions may include varying degrees of GEMS involvement. However, the objective of this study was not to isolate helicopter flight time or to quantify the extent of GEMS involvement during each mission. Rather, our aim was to evaluate whether the decision to use HEMS was justified from a system-level perspective, based on whether HEMS use was associated with a shorter total prehospital time to the actual receiving hospital. We have clarified this rationale in the Methods section and added the inability to model patient-level GEMS-HEMS operational processes as a limitation.

Attachment Submitted filename: Response_to_Reviewers.docx

# Editor report: Decision Letter 1

**DOI:** 10.1371/journal.pone.0353451.r003

**Authors:** Pawan Acharya, Pawan Acharya

25 Jun 2026

Assessing the appropriateness of helicopter emergency medical services for non-traumatic emergencies in a medically underserved rural area, Japan

PONE-D-26-10437R1

Dear Dr. Sada,

We’re pleased to inform you that your manuscript has been judged scientifically suitable for publication and will be formally accepted for publication once it meets all outstanding technical requirements.

Within one week, you’ll receive an e-mail detailing the required amendments. When these have been addressed, you’ll receive a formal acceptance letter and your manuscript will be scheduled for publication.

An invoice will be generated when your article is formally accepted. Please note, if your institution has a publishing partnership with PLOS and your article meets the relevant criteria, all or part of your publication costs will be covered. Please make sure your user information is up-to-date by logging into Editorial Manager at Editorial Manager® and clicking the ‘Update My Information' link at the top of the page. For questions related to billing, please contact billing support.

If your institution or institutions have a press office, please notify them about your upcoming paper to help maximize its impact. If they’ll be preparing press materials, please inform our press team as soon as possible -- no later than 48 hours after receiving the formal acceptance. Your manuscript will remain under strict press embargo until 2 pm Eastern Time on the date of publication. For more information, please contact onepress@plos.org.

Kind regards,

Pawan Acharya, PhD, MSPH

Academic Editor

PLOS One

Additional Editor Comments (optional):

Thank you for your thorough and thoughtful revision and for addressing the reviewers' comments in detail. The revisions have substantially improved the clarity of the manuscript, particularly regarding terminology, regional setting, and the rationale underlying the assessment of HEMS necessity.

Although some methodological limitations remain, particularly regarding the estimation of relative necessity based on historical prehospital times and the inability to account for all operational aspects of GEMS–HEMS interactions, these limitations are now appropriately acknowledged and discussed.

I believe the manuscript provides useful information that will be of interest to readers.

I am pleased to inform you that the manuscript is accepted for publication.

Reviewers' comments:

# Editor report: Acceptance letter

**DOI:** 10.1371/journal.pone.0353451.r004

**Authors:** Pawan Acharya, Pawan Acharya

PONE-D-26-10437R1

PLOS One

Dear Dr. Sada,

I'm pleased to inform you that your manuscript has been deemed suitable for publication in PLOS One. Congratulations! Your manuscript is now being handed over to our production team.

At this stage, our production department will prepare your paper for publication. This includes ensuring the following:

* All references, tables, and figures are properly cited

* All relevant supporting information is included in the manuscript submission,

* There are no issues that prevent the paper from being properly typeset

You will receive further instructions from the production team, including instructions on how to review your proof when it is ready. Please keep in mind that we are working through a large volume of accepted articles, so please give us a few days to review your paper and let you know the next and final steps.

Lastly, if your institution or institutions have a press office, please let them know about your upcoming paper now to help maximize its impact. If they'll be preparing press materials, please inform our press team within the next 48 hours. Your manuscript will remain under strict press embargo until 2 pm Eastern Time on the date of publication. For more information, please contact onepress@plos.org.

You will receive an invoice from PLOS for your publication fee after your manuscript has reached the completed accept phase. If you receive an email requesting payment before acceptance or for any other service, this may be a phishing scheme. Learn how to identify phishing emails and protect your accounts at https://explore.plos.org/phishing.

If we can help with anything else, please email us at customercare@plos.org.

Thank you for submitting your work to PLOS ONE and supporting open access.

Kind regards,

PLOS ONE Editorial Office Staff

on behalf of

Dr. Pawan Acharya

Academic Editor

PLOS One
